# Editorial: Participant characteristics and biological markers for endometriosis diagnosis or prediction of treatment response

**DOI:** 10.3389/frph.2024.1487745

**Published:** 2024-12-19

**Authors:** Amy L. Shafrir, Philippa T. K. Saunders

**Affiliations:** ^1^ Department of Health Sciences and Nutrition, School of Nursing and Health Sciences, Merrimack College, North Andover, MA, United States; ^2^ Boston Center for Endometriosis, Brigham and Women’s Hospital and Boston Children’s Hospital, Boston, MA, United States; ^3^ Division of Adolescent and Young Adult Medicine, Department of Pediatrics, Boston Children’s Hospital and Harvard Medical School, Boston, MA, United States; ^4^ Centre for Reproductive Health, Institute of Regeneration and Repair, The University of Edinburgh, Edinburgh, United Kingdom

**Keywords:** endometriosis, heterogeneity, biomarkers, complementary & alternative medicine, hormones

**Editorial on the Research Topic**
Participant characteristics and biological markers for endometriosis diagnosis or prediction of treatment response

Endometriosis, a common gynecologic disease affecting approximately 200 million women worldwide, and is associated with a range of symptoms, most commonly debilitating pelvic pain and infertility (1). Standard practice for a definitive endometriosis diagnosis has been through surgical visualization of endometriosis lesions. However, increasingly imaging is being used to inform symptom management strategies of certain endometriosis subtypes, particularly when fertility treatment is planned (1). The invasive nature of surgical diagnosis leads, in part, to the average 7-year delay between endometriosis symptom onset and diagnosis (2). Once a person is diagnosed with endometriosis, conventional treatments include the use of hormonal medications to suppress ovulation and surgical removal of the endometriotic lesions (1).

Adding to the complexity of the diagnostic delays and potential need for additional surgeries, is the extensive heterogeneity of endometriosis in symptom presentation and lesion characteristics (1). Endometriosis lesions vary in color, size, location, and histopathologic and metabolic characteristics, which may have important implications for treatment options. For example, a recent review highlighted the impact of fibrosis within endometriosis lesions, which may represent a potential future therapeutic target for endometriosis (3). Lesions with more fibrosis had higher numbers of nerve cells, which may be linked to increased pain symptoms (3). Other studies have suggested macrophages with altered phenotypes found in lesions are a potential therapeutic target and are the basis of a recently launched clinical trial [TCS/23/19, MacEndo] (4). However, further work is needed to expand on the range of trials based on targeting of lesion characteristics with full evaluation of their impact both on lesions and symptoms. Additionally, there does not exist a valid diagnostic tool with sufficient sensitivity and specificity to replace or to triage for diagnostic laparoscopic surgery. Further, no biological markers or algorithms exist for determining which endometriosis patients will experience recurrent symptoms and which patients will not.

The aim of this research topic was to further our understanding of endometriosis heterogeneity and how that heterogeneity may impact on biomarkers and/or algorithms for endometriosis diagnosis and treatment response.

Incorporating informative disease heterogeneity into biomarker analyses is critical for obtaining valid biomarker results (5). Two papers within this research topic investigated how heterogeneity among endometriosis participants may impact on endometriosis biomarker analyses. Although peritoneal fluid is often used to identify potential biological markers for endometriosis, peritoneal fluid heterogeneity in terms of color and volume in relation to patient characteristics has not been fully investigated (6, 7). Yousif et al. investigated participant characteristics that may differ by the color and volume of peritoneal fluid among 545 participants with endometriosis who had been enrolled in the Women's Health Study: from Adolescence to Adulthood cohort study (8). The authors found that peritoneal fluid color varied by menstrual cycle phase, hormonal medication use, and presence of acyclic pelvic pain. Additionally, peritoneal fluid volume appeared to differ by hormone medication use and presence of acyclic pelvic pain. These results suggest that accounting for menstrual cycle phase and hormone medication use in studies utilizing peritoneal fluid is important for informative biomarker discovery.

Additionally, in the last 5 years there has been increasing interest in utilizing microRNAs (miRNAs), small non-coding RNAs involved in epigenetic gene regulation, as biomarkers for endometriosis diagnosis (9), with some potentially promising results claimed for use of saliva as an accessible biofluid (10). In this special issue, Brady et al. investigated miRNAs in blood samples during adolescence and young adulthood based on measurements in a discovery cohort (10 cases and 10 controls) and internal replication (54 cases and 108 controls) group. During the discovery phase, 49 miRNAs were differentially expressed between endometriosis cases and controls. In the replication study, the miRNA score of miR-545-3p, let-7b-3p, miR-548i, miR-769-5p, and miR-30c-1-3p was found to have an area under the curve (AUC) of 0.77 (95% confidence intervals: 0.67–0.87). While this miRNA score was not sufficiently sensitive or specific enough to be used for an endometriosis diagnostic tool, the authors noted that expression of miRNAs among participants with and without endometriosis differed by hormone medication use. These results suggest that it is important to account for hormone medication use within studies of miRNAs, particularly as the vast majority of patients often use hormonal medications prior to their endometriosis diagnosis.

An important component of furthering our understanding of endometriosis heterogeneity is investigating new biomarkers to describe differences between endometriosis lesion subtypes to support development of more personalized approaches to therapy. To address this challenge, Zoet et al. developed a standardized protocol to assess nerve bundle density in endometriosis lesions among 236 endometriosis tissue samples from 121 patients. In this novel protocol, nerve bundle density was calculated as the number of nerve bundles that were immunopositive for PGP9.5 divided by the tissue surface area. The authors observed excellent intra- and inter-observer reliability for nerve bundle density and importantly found that nerve bundle density differed by endometriosis subtype. Higher nerve bundle density was noted for deep endometriosis and superficial lesions compared to endometriomas. These findings suggest that nerve bundle density may be a useful biomarker of local neurogenesis and has the potential to advance our understanding of how nerve bundle density differs by endometriosis subtypes and impacts on pathogenesis and treatment response of the disease.

Finally, up to 94% of endometriosis patients report utilizing complementary and/or alternative methods for coping with their endometriosis-associated pain (11), which includes growing interest in dietary modification (12). However, it is not known how the effectiveness of these coping strategies may differ by age group as past research has focused largely on adults with a confirmed endometriosis diagnosis. To address this gap in knowledge, Mongiovi et al. explored differences in use of complementary and alternative coping methods for acyclic pelvic pain symptoms among adolescents (age <18; *n* = 137), young adults (age 18–24; *n* = 143), and adults (age ≥25; *n* = 77). They observed that adolescents were more likely to report that sleeping (57%) and listening to music (21%) helped with their endometriosis-associated acyclic pelvic pain symptoms compared to young adults (44% and 7%, respectively) and adults (43% and 9%, respectively). Conversely, young adults (11%) and adults (15%) were more likely to report that yoga improved their acyclic pelvic pain compared to adolescents (4%). The impact of exercise on pain symptoms appeared to decrease with age, with 60% of adolescents, 47% of young adults, and 35% of adults reporting that exercise made their pain worse. These results highlight the need for developing personalized treatment plans for endometriosis patients that incorporate complementary and alternative approaches to pain management while taking into account the accessibility and acceptability of these approaches.

The papers included in this research topic highlight how heterogeneity in terms of patient characteristics and lesion characteristics have the potential to impact on the results of biomarker analyses and treatment acceptability. These papers offer a resource when considering the impact of heterogeneity on the design of future endometriosis research. Reflecting on how a greater appreciation of cancer heterogeneity has revolutionized knowledge and treatment options for cancers such as breast cancer (13), we believe an increase in data related to endometriosis heterogeneity has the potential to vastly expand our knowledge of the disease pathophysiology as well as aiding in the development of new diagnostics and treatment options.
